# Estimation of Ontogeny Functions for Renal Transporters Using a Combined Population Pharmacokinetic and Physiology-Based Pharmacokinetic Approach: Application to OAT1,3

**DOI:** 10.1208/s12248-021-00595-9

**Published:** 2021-05-04

**Authors:** Sînziana Cristea, Elke H. J. Krekels, Karel Allegaert, Peter De Paepe, Annick de Jaeger, Pieter De Cock, Catherijne A. J. Knibbe

**Affiliations:** 1 Division of Systems Biomedicine and Pharmacology, Leiden Academic Center for Drug Research, Leiden University, Leiden, The Netherlands; 2 Department of Development and Regeneration, KU Leuven, Leuven, Belgium; 3 Department of Pharmacy and Pharmaceutical Sciences, KU Leuven, Leuven, Belgium; 4 Department of Clinical Pharmacy, Erasmus MC, Rotterdam, The Netherlands; 5 Department of Pediatric Intensive Care, Ghent University Hospital, Ghent, Belgium; 6 Heymans Institute of Pharmacology, Ghent University, Ghent, Belgium; 7 Department of Pharmacy, Ghent University Hospital, Ghent, Belgium; 8 Department of Clinical Pharmacy, St. Antonius Hospital, Nieuwegein, The Netherlands

**Keywords:** Pediatrics, physiology-based PK, Population PK, OAT1,3, ontogeny

## Abstract

To date, information on the ontogeny of renal transporters is limited. Here, we propose to estimate the *in vivo* functional ontogeny of transporters using a combined population pharmacokinetic (popPK) and physiology-based pharmacokinetic (PBPK) modeling approach called popPBPK. Clavulanic acid and amoxicillin were used as probes for glomerular filtration, combined glomerular filtration, and active secretion through OAT1,3, respectively. The predictive value of the estimated OAT1,3 ontogeny function was assessed by PBPK predictions of renal clearance (CL_R_) of other OAT1,3 substrates: cefazolin and piperacillin. Individual CL_R_ *post-hoc* values, obtained from a published popPK model on the concomitant use of clavulanic acid and amoxicillin in critically ill children between 1 month and 15 years, were used as dependent variables in the popPBPK analysis. CL_R_ was re-parameterized according to PBPK principles, resulting in the estimation of OAT1,3-mediated intrinsic clearance (CL_int,OAT1,3,invivo_) and its ontogeny. CL_int,OAT1,3,invivo_ ontogeny was described by a sigmoidal function, reaching half of adult level around 7 months of age, comparable to findings based on renal transporter-specific protein expression data. PBPK-based CL_R_ predictions including this ontogeny function were reasonably accurate for piperacillin in a similar age range (2.5 months–15 years) as well as for cefazolin in neonates as compared to published data (%RMSPE of 21.2 and 22.8%, respectively and %PE within ±50%). Using this novel approach, we estimated an *in vivo* functional ontogeny profile for CL_int,OAT1,3,invivo_ that yields accurate CL_R_ predictions for different OAT1,3 substrates across different ages. This approach deserves further study on functional ontogeny of other transporters.

**Supplementary Information:**

The online version contains supplementary material available at 10.1208/s12248-021-00595-9.

## INTRODUCTION

Pediatric renal clearance (CL_R_) is driven by physiology related changes to kidney size, number of glomeruli and nephron filtration capacity, renal blood flow, expression of drug binding plasma proteins and expression of transporters. Throughout the pediatric age-range, the maturation of glomerular filtration rate (GFR) has been extensively studied by various groups (1–7), however, less is known about the functional *in vivo* development of other processes contributing to CL_R_ (8) such as active tubular secretion (ATS), which is mediated through transporters in the kidneys.

*In vivo* transporter activity cannot be directly quantified but has to be derived from other measures. Recently, the ontogeny of individual renal transporters has been quantified by measuring transporter-specific protein expressions in postmortem kidney samples from children of different ages (9). However, there is limited information about how protein expression relates to *in vivo* transporter activity and whether this relationship remains constant with age. Alternatively, ontogeny of ATS has been quantified *in vivo* as net secretion of drugs with non-selective affinity for transporters. Net secretion aggregates the activity of all active secretion transporters involved in renal excretion and of reabsorption (3, 10). Since ontogeny patterns may differ between transporters, their relative contributions to CL_R_ will also differ throughout the pediatric age-range, as drugs may have a broad spectrum in transporter affinity and can be transported by one or more transporters at once. Therefore, it would be of relevance to separately quantify the ontogeny of each renal transporter *in vivo*. Here we propose a new method to derive functional transporter ontogeny profiles *in vivo*.

Empirically, clinical pharmacokinetic (PK) data (i.e., concentration-time data) are analyzed using population PK (popPK) models. When analyzing pediatric PK data, the inter-individual variability in different parameters is driven by differences in underlying developing physiological processes. These differences are usually captured by a function that describes the relation between the individual deviations in parameter values from typical parameter values and a relatively small set of demographic variables that vary with age, i.e., covariate relationship. In pediatric physiology-based PK (PBPK) modeling, quantitative knowledge on developing physiology is included *a priori* in functions that describe changes in system-specific parameters. Subsequently, these models describe the interaction between drugs with certain physicochemical properties and this system. The parameters in a PBPK model can be derived from various data sources (e.g. *in vitro* experiments, clinical studies, etc.). Recently, combined popPK and PBPK approaches (which were referred to as popPBPK approaches, to not be confused with virtual PBPK populations) have been proposed to derive physiological measures for PBPK models that cannot be obtained through direct measures, by leveraging concentration-time data (11, 12). When selecting drugs that are predominantly eliminated by one main pathway, inferences can be made regarding system-specific parameters that are particular for that pathway.

In this study, the ontogeny of *in vivo* renal organic anion transporters 1 and 3 (OAT1,3) activity was characterized with this popPBPK approach. To this end, PK data obtained in critically ill children of different ages after the concomitant administration of clavulanic acid and amoxicillin was used. Each drug was assumed a probe for their specific elimination pathway, i.e., clavulanic acid for glomerular filtration (GF) and amoxicillin for a combination of GF and ATS through OAT1,3 (13, 14). With this methodology the ontogeny function of OAT1,3 could be estimated. Its predictive value was assessed by including the ontogeny function in a pediatric PBPK model to predict CL_R_ of two other OAT1,3 substrates including cefazolin and piperacillin.

## METHODS

### Software

For the present analysis we used NONMEM v7.3 integrated with Pirana v2.9.9 for developing the model and R v3.5 integrated with RStudio for graphics and evaluation.

### Quantifying the Ontogeny Function of OAT1,3 *In Vivo*

Clinical studies showed that the majority of an amoxicillin and clavulanic acid dose is recovered unchanged in urine (15–18) and *in vitro* evidence suggests that active secretion of amoxicillin is mainly mediated through OAT3 and to a lesser extent by OAT1 (13, 19). Different minor elimination routes may be involved, yet here we assume the clinical data to reflect the major elimination routes only. This implies the assumption that clavulanic acid clearance through other elimination routes than GF mature at the same rate as GF. For amoxicillin the extent of clearance through elimination routes other than active tubular secretion is assumed to be the same as for clavulanic acid and the difference in clearance between these two drugs is fully attributed to active tubular secretion through OAT1/3. Finally, even though the OAT1/3 transporter works in tandem with MRP4 efflux transporters, the contribution of MRP4 transporters to the CL_R_ of amoxicillin and for piperacillin and cefazolin, mentioned later in the “methods” section, was excluded in the current example as the expression of this transporter was found to remain constant with age (9).

Individual *post-hoc* CL_R_ values for clavulanic acid and amoxicillin in pediatric patients were obtained from a population PK model of De Cock *et al.* (20). In short, a simultaneous popPK analysis was performed for both drugs based on data obtained after the administration of a fixed dose ratio of 1:10 (clavulanic acid:amoxicillin) in 50 intensive care pediatric patients with ages between 1 month and 15 years (median age of 2.6 years) (20). The PK of clavulanic acid and amoxicillin were described by a two- and a three-compartment model, respectively, with inter-individual variability (IIV) on renal clearance (CL_R_) and central volume of distribution. The covariate analysis identified current weight as a statistically significant predictor for the IIV on both central volume of distribution and CL_R_, whereas vasopressor treatment and cystatin C were found to be statistically significant predictors only for the IIV on CL_R_ (20).

In a sequential step, CL_R_ was re-parameterized according to PBPK principles to reflect clearance through glomerular filtration (CL_GF_) and through active tubular secretion (CL_ATS_) (Eqs. 1 and 2) (21). The PBPK-based model for CL_R_ assumes a serial arrangement for GF and ATS, in which CL_R_ of clavulanic acid was described by CL_GF_ only (CL_ATS_ = 0), while CL_R_ of amoxicillin was described by a combination of CL_GF_ and CL_ATS_.

1 $$ C{L}_R=C{L}_{GF}+C{L}_{ATS}=\left( GFR\times {f}_u\right)+\left(\frac{\left({Q}_R- GFR\right)\times {f}_u\times C{L}_{\mathit{\sec}, OAT3}}{Q_R+ fu\times \frac{C{L}_{\mathit{\sec}, OAT3}}{BP}}\right) $$

2 $$ C{L}_{\mathit{\sec}, OAT3}=C{L}_{\mathit{\operatorname{int}}, OAT3, in\ vivo}\times on{t}_{OAT3}\times PTCPGK\times KW $$

In equation 1, GFR stands for glomerular filtration rate, f_u_ for drug fraction unbound, Q_R_ for renal blood flow, CL_sec,OAT1,3_ for secretion clearance through OAT1,3, and BP for blood to plasma ratio. Equation 2 shows how CL_sec,OAT1,3_ is obtained by multiplying CL_int,OAT1,3,in vivo_ that stands for OAT1,3-mediated *in vivo* intrinsic clearance in adults, with ont_OAT1,3_ that stands for the ontogeny function for OAT1,3, PTCPGK that stands for proximal tubule cells per gram kidney, and KW that stands for kidney weight in grams.

The adult PBPK-based model for CL_R_ through a combination of GF and ATS (Eqs. 1 and 2) was extrapolated to the pediatric population. For this, published functions that describe the age-related changes of the system-specific parameters (i.e., GFR (2), renal blood flow (22), and kidney weight (22)) and of the drug-specific parameters impacted by changes in system-specific parameters (i.e., serum albumin concentrations (4) that influence the fraction unbound (23), and hematocrit levels that influence BP (22)) were inputted, as shown in Table S1. Values for f_u_ (24) and BP_amox ._(25) as reported in adults were used (f_u clav.acid_ = 0.75; f_u amox_ = 0.82; BP_amox._= 0.55). CL_int,OAT1,3,in vivo_ reflects both the expression and activity of the OAT1,3 transporter in adults. Assuming PTCPGK to remain constant at adult values, this only leaves CL_int,OAT1,3,in vivo_ and its ontogeny function (ont_OAT1,3_) to be estimated. This was done using the individual CL_R_ values from the population model as dependent variables and deriving the system-specific PBPK parameters based on the individual patient characteristics for each patient.

Pediatric typical CL_GF_ values were obtained using a published GFR maturation function developed for children with a normal renal function (2). However, when compared to normal CL_GF_ values, CL_R_ of both drugs as estimated with the population PK models, were found to be increased in the critically ill children included in the dataset of the current analysis (20). Hence, the PBPK-based re-parameterization of CL_GF_ included a typical GF correction factor (θ_corr_) with IIV (ƞ_GFR_) to account for this difference (equations 3).

3 $$ C{L}_{R, clavulanic\ acid,i}= GFR\times {f_u}_{\boldsymbol{clav}.\kern0.5em \boldsymbol{acid}}\times {\theta}_{corr.}\times {e}^{\eta_{GFR}} $$

As both amoxicillin and clavulanic acid were administered simultaneously to each child, from the data on clavulanic acid the GF correction factor and IIV on GFR for each pediatric patient was estimated. According to Eqs. 4 and 5, the difference between the individual values for CL_R_ of amoxicillin and CL_R_ of clavulanic acid were used to estimate CL_ATS_, which was the basis for the estimation of the IIV on the *in vivo* CL_sec,OAT1,3_ value and subsequently the OAT1,3 ontogeny function (ont_OAT1,3_).

4 $$ C{L}_{\mathrm{R}, amoxicilin,i}= GFR\times {f_u}_{\boldsymbol{amox}.}\times {\theta}_{corr.}\times {e}^{\eta_{GFR}}+\frac{\left({Q}_R- GFR\right)\times {f_u}_{amox.}\times C{L}_{\mathit{\sec}, OAT1,3,i}}{Q_R+{f_u}_{amox.}\times \frac{C{L}_{\mathit{\sec}, OAT1,3,i}}{B{P}_{amox.}}} $$

5 $$ C{L}_{\mathit{\sec}, OAT1,3,i}={\theta}_{CLint, OAT1,3, in\ vivo}\times {e}^{\eta_{CLint, OAT3, in vivo}}\times on{t}_{OAT1,3}\times PTCPGK\times KW $$

To quantify the ontogeny profile of CL_int,OAT1,3_,_in vivo_, different covariates (i.e. postnatal age, postmenstrual age, weight) were explored using sigmoid relationships (Eq. 6) or a simplification of this equation (i.e., an exponential equation). In Eq. 6, *hill* is the hill coefficient, which quantifies the steepness of the ontogeny slope and *TM*_*50*_ quantifies the age at which OAT1,3 reaches half of the adult value.

6 $$ on{t}_{OAT1,3}=\frac{CO{V}^{hill}}{CO{V}^{hill}+T{M}_{50}^{hill}}, $$

The statistical significance of including the ont_OAT1,3_ function in the equation for CL_sec,OAT1,3,i_ to obtain CL_R_ of amoxicillin was assessed according to the likelihood ratio test on the difference in objective function value. Under the assumption of a χ^2^ distribution, the objective function value of a model with one more degree of freedom had to be 3.84 points lower, with a corresponding *p* < 0.05 to indicate statistical significance (26). For graphical goodness-of-fit, a plot was made to check for prediction bias of the individual CL_R_ values obtained either with the PBPK model or the individual *post hoc* values from the population PK model that served as the dependent variable in these fits. In addition, ETA (ƞ_GFR_, ƞ_CLint,OAT1,3,invivo_) vs. covariate plots (age, weight) are made to check for structural accuracy in PK parameters.

### Predictive Properties of the OAT1,3 Ontogeny Function for New Substrates

To assess the predictive performance of the obtained OAT1,3 maturation function, the PBPK model that includes the estimated ontogeny function for OAT1,3 (Eqs. 1 and 2) was used for pediatric PBPK CL_R_ predictions of piperacillin and cefazolin, two other substrates of the OAT1,3 transporter. PBPK predictions of CL_R_ were compared to published typical pediatric CL_R_ predictions by population PK models of the same drugs. Population models are considered the gold standard for deriving CL_R_ values from observed concentration-time data and since neither the PBPK model nor the typical predictions by a population PK model take random inter-individual deviations in CL_R_ into account, they can be directly compared.

To obtain the pediatric PBPK predictions for CL_R_, we collected literature values for f_u, adult_ of 0.8 (27) and 0.31 (25) for piperacillin and cefazolin, respectively, and for BP _adult_ of 0.55 for both drugs. CL_int,OAT1,3,in vivo_ in Eq. 2 had to be derived for both drugs. This was done based on published *in vitro* activity data as measured in assays with OAT1,3 transfected cells (1.95 μl/min/mg protein (27) and 7.1 μl/min/mg protein (25) for piperacillin and cefazolin respectively). These values were further optimized based on the *in vivo* adult values for CL_int,OAT1,3_ using a retrospective IVIVE approach. More details on the retrospective IVIVE are provided in the supplemental materials.

The drug-specific CL_int,OAT1,3, in vivo_ values obtained in the retrospective IVIVE step were used in Eqs. 1 and 2 of the renal PBPK model to obtain pediatric CL_R_ predictions for cefazolin and piperacillin. Pediatric PBPK CL_R_ predictions for piperacillin and cefazolin were made for typical individuals with the same demographic characteristics as the individual patients reported in the original publications describing the pediatric population PK models of these drugs (20, 28). This means that, for piperacillin, PBPK CL_R_ values were estimated for 47 pediatric patients with ages between 2.5 months and 15 years (median age of 2.83 years). For cefazolin, the PBPK CL_R_ values were estimated for 26 near-term neonates with gestational age higher than 35 weeks and postnatal age (PNA) between 1 and 30 days (median of 8 days). For this, the OAT3 ontogeny function obtained above for children of 1 month and older based on data from clavulanic acid and amoxicillin was extrapolated to the neonatal population.

Pediatric PBPK CL_R_ predictions were visually and quantitatively compared to typical estimates obtained with published population PK models for these two OAT1,3 substrates. Precision was quantified as percentage root mean square prediction error (%RMSPE) (Eq. 7) and bias as percentage prediction error (%PE) (Eq. 8).

7 $$ \% RMSPE=\sqrt{\frac{1}{N}\times \sum \limits_{i=1}^N{\left(\frac{C{L_{R,}}_{PBPK}-C{L_{R,}}_{reference}}{C{L_{R,}}_{reference}}\right)}^2}\times 100, $$

8 $$ \%\mathrm{PE}=\left(\frac{C{L_{R,}}_{PBPK}-C{L_{R,}}_{reference}}{C{L_{R,}}_{reference}}\right)\times 100, $$

In both equations, *CL*_*R,PBPK*_ are the CL_R_ predictions obtained with the renal PBPK model in pediatrics and *CL*_*R,reference*_ represents the CL_R_ values for typical CL_R_ predictions obtained with the published population PK models (28, 29). %RMSPE and %PE were calculated separately for piperacillin and cefazolin and reported overall as well as per age group. CL_R,PBPK_ was considered to be accurately predicted if %RMSPE and %PE was within ±30%, reasonably accurately predicted between −30–−50% and 30–50% and inaccurate when %RMSPE and %PE were outside ±50%. Note that %RMSPE can only take positive values.

## RESULTS

### Quantifying the Ontogeny Function of OAT1,3

**Fig. 1:**
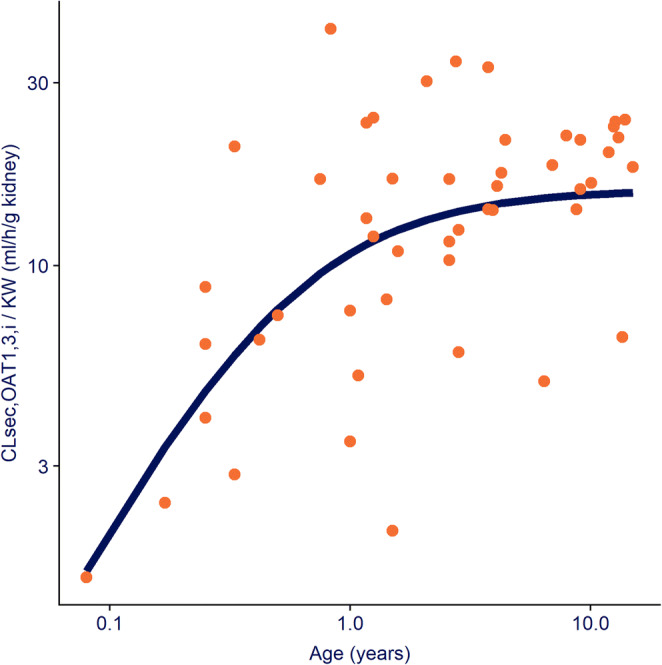
Ontogeny function for OAT1,3-mediated intrinsic clearance normalized by kidney weight (CL_sec,OAT1,3_– blue line) described by a sigmoidal function based on age and displayed throughout the studied pediatric age-range (1 month to 15 years), on a double-log scale. The orange dots represent the individual secretion clearance estimates normalized by kidney weight. See Eq. [5] for more details

With the popPBPK approach, CL_GF_ was separated from CL_ATS_ such that CL_int,OAT1,3,in vivo_ and its ontogeny profile could be estimated in children as young as 1 month up to 15 years of age. Figure 1 shows the ontogeny profile of OAT1,3 as best described by a sigmoidal relationship based on PNA. CL_int, OAT1,3, in vivo_ was estimated to be 15.8 ml/h/g kidney (RSE% of 5%) at 15 years with an IIV of 78.5%. This high IIV suggests large differences between individual values obtained for CL_int, OAT1,3, in vivo_. CL_int, OAT1,3, in vivo_ was found to reach half of the adult capacity at a PNA of 27.3 weeks (RSE of 28%), which is around 7 months. The rapid ontogeny of OAT1,3 was captured by a *hill* exponent of 1.17 (%RSE of 36%). The estimated transporter ontogeny fractions range from 0.1 at 1 month and 1 at 15 years. The GF correction factor used to account for the increased CL_R_ in critically ill children was estimated at 1.83 (RSE of 4%) with an IIV of 24.4%.

The goodness-of-fit plots did not show any bias for CL_R_ predictions obtained with CL_R_ re-parameterized according to PBPK principles. Neither Fig. S1, which depicts popPBPK CL_R_ predictions vs. the popPK CL_R_ predictions, nor Fig. S2, which depicts the ƞ_GFR_ and ƞ_CLint,OAT1,3,in vivo_ vs. covariates (i.e., weight and age) show any bias. This suggests that the PBPK-based re-parameterization as CL_GF_ (Eq. 3) can predict individual clavulanic acid CL_R_ values accurately and that the reparameterization for CL_GF_ together with CL_ATS_ (Eq. 4) can accurately predict the CL_R_ of amoxicillin as excreted by GF and ATS through OAT1,3.

**Fig. 2:**
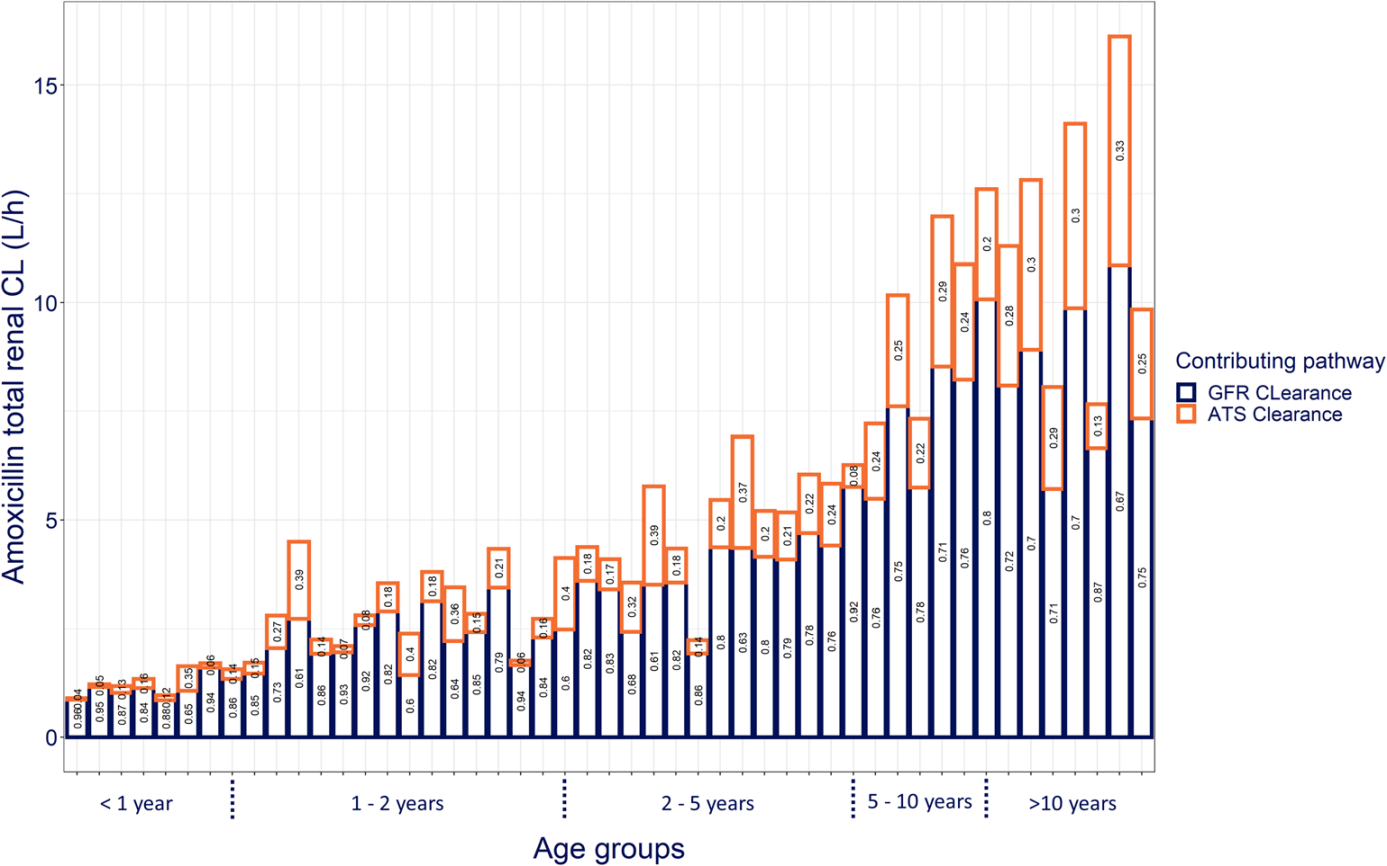
Contribution of clearance through glomerular filtration (CL_GF_ – bottom blue boxes) and through active tubular secretion (CL_ATS_ – top orange boxes) to total renal clearance of amoxicillin (CL_R_ – sum of blue and orange boxes) for each pediatric patient of the studied population sorted and grouped by age. The numbers in each box show the relative contribution of CL_GF_ and CL_ATS_ to total CL_R_ for each individual

Figure 2 shows the total CL_R_ for amoxicillin and the contribution of CL_GF_ and CL_ATS_ to CL_R_ for each individual. Total CL_R_ increases almost 7-fold between neonates younger than 1 year and children of 10 years and older (median of 1.64 L/h and 12 L/h, respectively). The median contribution of ATS to amoxicillin CL_R_ for the studied pediatric population was 22% (range: 4–40%). Even if variability in ATS contribution was high within groups of individuals with similar ages, the ATS contribution increased with age, on average, from 14% in children younger than 1 year to 18% in children of 1–2 years, 21% for children of 2–5 years, 24% for children 5–10 years, reaching 29% for children older than 10 years.

### Predictive Properties of the OAT1,3 Ontogeny Function

**Fig. 3:**
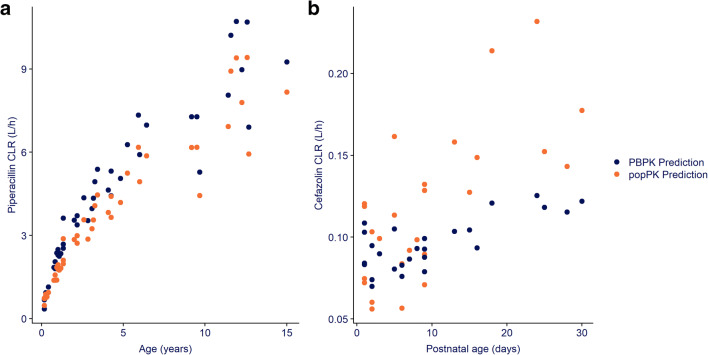
Renal clearance (CL_R_) of piperacillin (**a**) and cefazolin (**b**) versus age in pediatric patients in children (**a**) and neonates (**b**). The pediatric PBPK CL_R_ predictions (dark blue) are overlaid with the typical CL_R_ estimates obtained with the published population pharmacokinetic model (orange)

Figure 3 shows the pediatric CL_R_ predictions for piperacillin and cefazolin obtained with the PBPK-based model and the identified OAT1,3 ontogeny function based on clavulanic acid and amoxicillin overlaid with the typical clearance estimates obtained with the published population PK models. The %RMSPE calculated between PBPK CL_R_ and typical CL_R_ predictions for piperacillin (Fig. 3a) over the entire age-range (2.5 months to 15 years) was 21.8% with a %PE interval between −33.2% and 25.4%. When stratified per age groups (i.e., younger than 1 year, 1–2 years, 2–5 years, 5–10 years and older than 10 years) %RMSPE is generally higher for children under 5 years (23.3, 22.2, and 27.4% vs. 14.9, 18.8%). For neonates (Fig. 3b), the %RMSPE calculated between PBPK CL_R_ and typical CL_R_ predictions for cefazolin was 22.2% with %PE interval between −34.4 and 46%.

For both pediatric populations the PBPK-based CL_R_ predictions can be considered reasonably accurate with %RMPE < 30% and %PE within ±50%. For piperacillin, the PBPK-based CL_R_ predictions tend towards overprediction (Fig. 3a), with all %PE values below 0%, although percentage deviations were acceptable [%PE between −13.3 and −28.8%] for children older than 1 year. For cefazolin in neonates, predictions are reasonably accurate (Fig. 3b), with PBPK-based CL_R_ predictions tending towards underprediction [%PE between 18.1 and 46%] for children older than 10 days.

## DISCUSSION

With a combined population PK with PBPK approach, referred to as popPBPK, we estimated the functional *in vivo* ontogeny profile for OAT1,3, a parameter that cannot be obtained through direct measurements, down to the age of 1 month. Under the assumption that clavulanic acid is entirely eliminated through GF and amoxicillin through GF and ATS through OAT1,3, we used clinical PK data of children that received both drugs at the time to define a maturation function for ATS through OAT1,3. Using a population PK approach, we derived the individual CL_R_ values for both drugs that served as dependent variable for the popPBPK approach. CL_R_ was re-parameterized according to PBPK principles to take advantage of existing information about drug- and system-specific properties while estimating the ontogeny of OAT1,3 *in vivo* and the variability on GFR and on OAT1,3-mediated intrinsic clearance *in vivo* (CL_int,OAT1,3, in vivo_).

Our group recently developed a PBPK simulation framework for investigating the impact of ontogeny of renal secretion transporters on CL_R_ by predicting pediatric CL_R_ for hypothetical drugs with an array of drug properties (30). By looking at the difference between PBPK CL_R_ predictions with or without inclusion of the ontogeny function, probe drugs for quantifying the ontogeny of transporters were identified. According to the findings with this framework, amoxicillin, which has an estimated CL_int,OAT1,3, in vivo_ of 4.4 μl/min/mg protein and a f_u_ of 0.82 (31), has the potential of serving as a probe to quantify OAT1,3 ontogeny. Furthermore, the clinical data available for probe drugs for GF and a combination of GF and ATS (clavulanic acid and amoxicillin, respectively) administrated to the same individuals was paramount to separate between these two processes.

OAT1,3 ontogeny for the OAT1,3-mediated intrinsic clearance is steep in the first year of life, attaining half of the adult value around 7 months of age. This estimated ontogeny function was included in the pediatric PBPK-based model for CL_R_ through GF and ATS. Even though the functional *in vivo* OAT1,3 ontogeny profile was derived from clinical data obtained in critically ill patients without renal disfunction, it predicted the CL_R_ for other drugs that are substrates for OAT1,3 reasonably accurate, as compared to popPK CL_R_ predictions for these drugs. Assuming clearance to be only mediated by GF and ATS, for piperacillin the PBPK CL_R_ predictions over an age-range of 2.5 months to 15 years lead to a %RMSPE of 21.8% [%PE: −33.2–25.4%] with a trend towards over-prediction for children older than 1 year. For cefazolin, extrapolation of CL_R_ predictions to near term neonates with ages between 1- and 30-days lead to a %RMSPE of 22.2% [%PE: −34.4%–46%.], with a trend towards under-prediction for children older than 10 days.

**Fig. 4:**
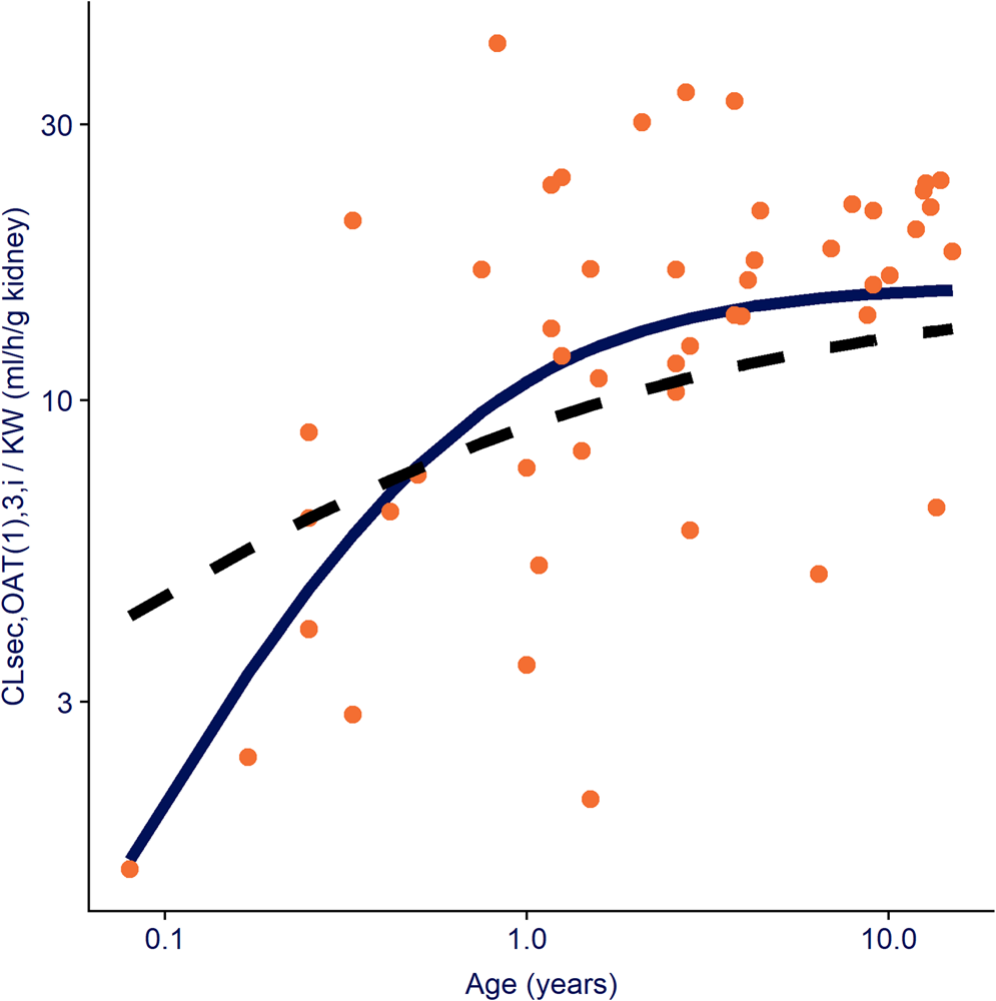
Ontogeny functions for OAT(1),3-mediated intrinsic clearance normalized by kidney weight (CL_int,OAT1,3,in vivo_) throughout the studied pediatric age-range (1 month to 15 years). The solid line shows the sigmoidal function estimated in the current analysis whereas the dashed line shows the ontogeny function for OAT1 as published by Cheung (9)*.* The orange dots represent the individual secretion clearance estimates normalized by kidney weight derived from amoxicillin CL_R_ values obtained with the current analysis. See Eq. [5] for more details

Previously, Hayton *et al.* used para-aminohippurate to derive an ontogeny profile of undifferentiated active renal secretion *in vivo*, concluding that 50% maturation is achieved around 1 year of age (3), which is comparable with our findings. Recently, more insight into differentiated ontogeny profiles of individual renal transporters have been quantified based on direct measurements of the expression of transporter-specific proteins in kidney samples taken postmortem from children of various ages, as described in detail by Cheung *et al.* (9). This group characterized the ontogeny of OAT1,3 as a sigmoidal function based on PNA in weeks with children reaching half of the adult values around 8 months of age (TM_50_ = 30.7 weeks [95% CI: 16.64–50.97]) and the steepness of the ontogeny slope given by a *hill* coefficient of 0.51 (95% CI: 0.35–0.71). While our findings align with Cheung *et al*. regarding the age at which half of the adult level is reached, which was estimated to be around 7 months with our function, we found a steeper ontogeny for OAT1,3, as shown by a 2-fold higher estimated *hill* coefficient. The impact of these differences on the ontogeny profiles is illustrated in Fig. 4. This figure shows relatively similar OAT1,3 ontogeny found by both methods at ages above the TM_50_ values, but for younger ages the function quantified in our work shows lower ontogeny values. Given the low number of observed values at these younger ages in both analyses, the uncertainty around the ontogeny below 7 months of age is high for both analyses. More data are required to establish the accuracy of the estimated *in vivo* functional ontogeny profile in the first year of life.

Although drugs may be predominantly eliminated by a particular pathway, they are rarely exclusively cleared through a single, well-defined pathway. However, despite the fact that the estimated functional OAT1,3 ontogeny profile may be impacted by minor elimination pathways contributing to the clearance of clavulanic acid and amoxicillin, this function could be used to obtain accurate pediatric PBPK-based CL_R_ predictions for two other drugs that are predominantly, though not exclusively, eliminated through GF and OAT1,3-mediated ATS, namely piperacillin and cefazolin. Despite small trends towards over and under-prediction respectively, CL_R_ predictions for piperacillin and cefazolin were reasonably accurate with %RMSPE of 21.8 and 22.2%, which is well below the 2-fold error, which is the generally accepted criterion for accuracy of PBPK predictions. The tendency towards over-prediction of pediatric PBPK CL_R_ for piperacillin could be explained by other processes involved in renal elimination that are not accounted for in the PBPK model. It could for instance be that there is passive or active reuptake of these drugs in the kidneys. Alternatively, the authors of the popPK model that served as the reference values, reported a (temporary) impairment of the renal maturation function (29) which could explain the lower CL_R_ values obtained with the popPK model as compared to the PBPK CL_R_ predictions, the latter of which does not take (potential) renal impairment into account. A second drug, cefazolin, was used to assess the accuracy of this function for extrapolations to term newborns below 1 month of age. Remarkably, despite a small trend towards under-prediction of CL_R_ values for cefazolin in part of the newborns, all predictions can still be considered accurate.

The methodology proposed here is the first to enable the assessment of functional *in vivo* activity, rather than mRNA or transporter expression or *ex vivo* activity. As such it cannot only augment the currently available methods to study renal transporter maturation throughout the pediatric age-range, but can also offer a valuable new dimension to this research. Essential in our approach is the requirement of data on two probe drugs that are predominantly excreted by specifically GFR and a combination of GFR and ATS through a specific transporter. As studies in healthy pediatric populations are not allowed, the two probe drugs would have to be regularly prescribed for therapeutic purposes in children across the entire age-range. Furthermore, practical and ethical constraints may require assumptions to be made in the implementation of this method. For instance, in the example used here to illustrate our approach, we assumed exclusive elimination of our probe drugs through GF and OAT1,3-mediated ATS, as ethical and practical constraints prevent urine collection over prolonged durations and thereby the formal assessment of the contribution of renal excretion to overall drug elimination. This may have impacted the accuracy of the obtained ontogeny function, but it does not impact our proposed methodology conceptually. If information on minor elimination routes would become available, this could be included in the PBPK model to further refine the estimated ontogeny function.

## CONCLUSION

The ontogeny of functional *in vivo* OAT1,3 activity was derived by using a combined population PK and PBPK modeling approach. This popPBPK approach leverages the knowledge on underlying physiological processes included in PBPK models and information carried by individual PK parameters as quantified with a population approach, to derive parameters that cannot be measured *in vivo.* With this methodology we derived the renal OAT1,3 transporter ontogeny *in vivo*. This ontogeny function was included in the pediatric PBPK-based model CL_R_ for two other OAT1,3 substrates and on average predicted CL_R_ throughout the entire pediatric age-range accurately. This methodology could be applied to other transporters substrates to characterize the *in vivo* ontogeny of the remaining renal transporters to further increase our understanding on renal development and increase the accuracy in predicting pediatric CL_R_.

## Supplementary Information

ESM 1 (DOCX 511 kb)
