# Supplementary material for: Estimation of Ontogeny Functions for Renal Transporters Using a Combined Population Pharmacokinetic and Physiology-Based Pharmacokinetic Approach: Application to OAT1,3
Source: AAPS J. 2021 May 4;23(3):65. doi: 10.1208/s12248-021-00595-9 (PMC8096729; doi:10.1208/s12248-021-00595-9)
Supplement: Supplementary file 1 — (DOCX 511 kb) [file 12248_2021_595_MOESM1_ESM.docx]

**Supplemental material**

**Estimation of ontogeny functions for renal transporters using a combined population pharmacokinetic and physiology-based pharmacokinetic approach: application to OAT1,3**

Sînziana Cristea^1^, Elke Henriëtte Josephina Krekels^1^, Karel Allegaert^2,3,4^, Peter De Paepe^5^, Annick de Jaeger^6^, Pieter De Cock^5,6,7^, Catherijne Annette Jantine Knibbe^1,8^

^1^Division of Systems Biomedicine and Pharmacology, Leiden Academic Center for Drug Research, Leiden University, Leiden, The Netherlands

^2^Department of Development and Regeneration, KU Leuven, Belgium

^3^Department of Pharmacy and Pharmaceutical Sciences, KU Leuven, Belgium

^4^Department of Clinical Pharmacy, Erasmus MC, Rotterdam, The Netherlands

^5^Department of Pediatric Intensive Care, Ghent University Hospital, Belgium

^6^Heymans Institute of Pharmacology, Ghent University, Belgium

^7^Department of Pharmacy, Ghent University Hospital, Ghent, Belgium

^8^Department of Clinical Pharmacy, St. Antonius Hospital, Nieuwegein, The Netherlands

Table S1 –Functions describing age-related changes of system-specific parameters and variables required by the PBPK model for pediatric CL_R_ predictions

| System-specific parameters for equation [1] (abbreviation)  [units] | Maturation functions included in the pediatric PBPK model for CL_R_ | | | | References |
| --- | --- | --- | --- | --- | --- |
| Glomerular filtration rate (GFR)  [ml/min] | $GFR= 112 \times\left( \frac{WT}{70} \right)^{0.63}\times\left( \frac{PMA^{3.3}}{PMA^{3.3}+{55.4}^{3.3}} \right)$ | | | | (1) |
| Fraction unbound  (f_u_)  [-] | $\left[ HSA \right]_{ped/adult}=1.1287\times\ln\left( AGE \right)+33.746$  $f_{u,clav. acid}=0.75;f_{u,amox.}=0.82 (adult values)$  $\boldsymbol{\to}\boldsymbol{f}_{\boldsymbol{u}\boldsymbol{,}\boldsymbol{ped}}\boldsymbol{=}\frac{\boldsymbol{1}}{\boldsymbol{1}\boldsymbol{+}\frac{\left( \boldsymbol{1}\boldsymbol{-}\boldsymbol{f}_{\boldsymbol{u}\boldsymbol{,}\boldsymbol{drug}} \right)\boldsymbol{\times}\left[ \boldsymbol{HSA} \right]_{\boldsymbol{ped}}}{\left[ \boldsymbol{HSA} \right]_{\boldsymbol{adult}}\boldsymbol{\times}\boldsymbol{f}_{\boldsymbol{u}\boldsymbol{,}\boldsymbol{drug}}}}$ | | | | (2) |
| Renal blood flow  (Q_R_)  [ml/min] | $CO=BSA\times\left( 110+184\times e^{-0.0378\times AGE}-e^{-0.24477\times AGE} \right)$  $fr=\frac{fr_{males}+fr_{females}}{2}$  $fr_{males}=4.53+\left( 14.63\times\frac{AGE}{0.1888+AGE} \right)$  $fr_{females}=4.53+\left( 13\times\frac{{AGE}^{1.15}}{{0.188}^{1.15}+{AGE}^{1.15}} \right)$  $\boldsymbol{\to}\boldsymbol{Q}_{\boldsymbol{R}}\boldsymbol{=CO\times fr}$ | | | | (2) |
| Intrinsic secretion CL  (CL_sec,OAT1,3_)  [mL/min] | $PTCPKG=60 (adult value)$  $KW=1050\times\left( 4.214\times WT^{0.823}+4.456\times WT^{0.795} \right)/1000$  $\boldsymbol{\to} \boldsymbol{C}\boldsymbol{L}_{\boldsymbol{sec,OAT}\boldsymbol{1,3}}\boldsymbol{=C}\boldsymbol{L}_{\boldsymbol{int,OAT}\boldsymbol{1,3,in vivo}}\boldsymbol{\times on}\boldsymbol{t}_{\boldsymbol{OAT}\boldsymbol{1,3}}\boldsymbol{\times PTCPGK\times KW}$ | | | | (2) |
| Blood to plasma ratio  (BP_amoxicillin_)  [-] | $hemat=\frac{hemat_{male}+hemat_{female}}{2}$  $hemat_{male}=53-\left( \left( 43\times\frac{{AGE}^{1.12}}{{0.05}^{1.12}+{AGE}^{1.12}} \right)\times\left( 1+\left( -0.93\times\frac{{AGE}^{0.25}}{{0.10}^{0.25}+{AGE}^{0.25}} \right) \right) \right)$  $hemat_{female}=53-\left( \left( 37.4\times\frac{{AGE}^{1.12}}{{0.05}^{1.12}+{AGE}^{1.12}} \right)\times\left( 1+\left( -0.80\times\frac{{AGE}^{0.25}}{{0.10}^{0.25}+{AGE}^{0.25}} \right) \right) \right)$  $\to\boldsymbol{BP =1+hemat\times}\left( \boldsymbol{f}_{\boldsymbol{u}}\boldsymbol{\times}\boldsymbol{k}_{\boldsymbol{p}}\boldsymbol{-1} \right)$ | | | | (2) |
| **WT** – bodyweight [kg]  **PMA** – postmenstrual age [weeks], this is derived from gestational age and postnatal age, in which gestational age was assumed to be 40 weeks when individual values were unknown.  **[HSA]** – human serum albumin [g/L]  **CO** – cardiac output [mL/min]  **hemat** – hematocrit  **fr** – fraction of cardiac output directed to renal artery  **AGE** – age in [days] for the maturation of [HSA] and in [years] for the fraction of cardiac output and hematocrit levels | | **BSA** – body surface area (m^2^)  **PTCPGK** – proximal tubule cells per gram kidney [x 10^6^ cells]  **KW** – kidney weight [g]  **ont_OAT1,3_** – OAT1,3 ontogeny relative to adult levels [-]  **CL_int_**_,OAT1,3_ – OAT1,3-mediated active clearance [ml/min]  **k_p_** – blood-to-plasma partitioning coefficient of a drug |  |  |  |

Retrospective *in vitro – in vivo extrapolation (IVIVE)*

The CL_int,OAT1,3,in vivo_ values required for the PBPK-based model for CL_R_ (equation 1 and 2 of the main document), were obtained following retrospective *in vitro-in vivo extrapolation* as shown in equation S1. Published *in vitro* values for OAT3-mediated intrinsic clearance (CL_int,OAT3, in vitro_) for piperacillin and cefazolin (3)^,^(4) obtained from tissue samples from adults (Table S2), were extrapolated to CL_int,OAT1,3, in vivo_ based on the protein expression correction factor (relative active factor (RAF)) between the OAT-transfected cells in the *in vitro* assay and the in proximal tubule cells and an activity adjustment factor (AAF).

$CL_{int,OAT1,3,in vivo}=CL_{int,OAT3, in vitro}\times protein correction\times RAF\times AAF$, [S1]

In equation S1, *protein correction* represents the total amount of proteins in 10^6^ cells obtained from the *in vitro* sample, under the assumption that 10^6^ cells from this sample is equivalent to 10^6^ proximal tubule cells in the kidney. *RAF* is an activity correction factor between the *in vitro* and the *in vivo* OAT1,3 transporter activity. These first two parameters are specific to the *in vitro* assay and independent of the studied drug. AAF is the activity adjustment factor, which is included as a correction factor for CL_int,OAT3,in vitro_ to account for the discrepancy between the CL_R_ obtained with adult PBPK model and the reported CL_R_ values in literature (5).

For performing the retrospective IVIVE, a protein expression value of 0.25 mg protein per 10^6^ Human Embryonic Kidney 293 (HEK293) OAT-transfected cells was used as measured and reported by Mathialagan (4) for their uptake assay(4). For cefazoline, CL_int,OAT3,in vitro_ was measured by the same group, whereas for piperacillin this value was obtained from a similar system but developed by another research group (Wen *et al.* (3)). Since the protein expression value is not reported for Wen *et al.* (3), the protein expression value was assumed to be the same between cell systems and included as such for the IVIVE.

The RAF value used for OAT3 was previously determined by Mathialagan (4) by using selective substrates for OAT transporters to account for the difference between the scaled *in vitro* and *in vivo* intrinsic secretion clearance (CL_sec,OAT_ – equation 2 of the main document). For OAT3, the reported value was 4.6 and this drug-independent value was included as such in equation S1.

AAF was obtained by back-calculation to match the literature values collected for CL_R_. Using the literature adult CL_R_, the PBPK model was then solved for CL_sec,OAT1,3_ as shown in equation S2A. The result was used in equation S3A, which was solved for CL_int,OAT1,3, in vivo._ The obtained CL_int,OAT1,3, in vivo_ was used as input in equation S4A, solved for AAF. This factor was then multiplied with the relevant parameter to obtain the *in vivo* OAT1,3-mediated intrinsic clearance. AAF accounts for any activity differences between *in vitro* assays and *in vivo* derived activity in adults (5).

$CL_{R,lit.}=f_{u}\times GFR+\frac{\left( Q_{R}-GFR \right)\times f_{u}\times CL_{sec,OAT1,3}}{Q_{R}+f_{u}\times\frac{CL_{sec,OAT1,3}}{BP}}$ [S2]

${CL}_{sec,OAT1,3}=\frac{\left( CL_{R,lit.}-f_{u}\times GFR \right)\times Q_{R}}{({(Q}_{R}-GFR)\times f_{u} -(CL_{R}-f_{u}\times GFR)\times\frac{fu}{BP})}$ [S2A]

$CL_{sec,OAT1,3}=ont_{OAT1,3}\times CL_{int,OAT1,3}\times PTCPGK\times KW$ [S3]

$CL_{int,OAT1,3}=\frac{CL_{sec,OAT1,3}}{ont_{OAT1,3}\times PTCPGK\times KW}$ [S3A]

$CL_{int,OAT1,3,in vivo}=CL_{int,OAT1,3, in vitro}\times protein expression\times RAF\times AAF$, [S4]

$AAF=\frac{CL_{int,OAT1,3,in vivo}}{CL_{int, OAT1,3, in vitro}\times protein expression\times RAF}$ [S4A]

This retrospective IVIVE procedure quantifies the AAF that corrects for known unknowns and unknown unknowns in the PBPK model, to optimize the PBPK-based CL_R_ predictions such that this yields an optimized fit of the observed CL_R_ in adults. The subsequent scaling from AAF-corrected adult CL_R_ to pediatric CL_R_ is exclusively based on the PBPK predictions, without parameter fitting/corrections. This allows us to purely assess this scaling accuracy, while taking out potential bias resulting from unknowns in translating in vitro measures to in vivo functional CL_R_ predictions.

The drug-specific parameters required as input for the PBPK-based model (i.e. f_u_ and BP) were collected from literature for each drug(6,7) (Table S2). Literature values of adult CL_R_ for cefazolin and piperacillin were collected together with the reported median values of the demographic characteristics in these reports (i.e. weight, age) (Table S2), as these values were needed to derive the system-specific parameters required in equations S2 and S3 (i.e. GFR, Q_R_, KW, HSA concentration). As the PBPK model is for adults, ont_OAT3_ was fixed at the adult level (ont_OAT1,3_ = 1). The CL_int,OAT1,3,in vivo_ obtained after the retrospective IVIVE step in adults was included in the pediatric PBPK model for CL_R_.

Table S2 – Adult demographic characteristics and drug-specific parameters used for PBPK-based CL_R_ predictions as well as published typical CL_R_ values for adults as obtained with popPK models, for piperacillin and cefazolin.

|  | | Piperacillin  value [unit] | Cefazolin  value [unit] |
| --- | --- | --- | --- |
| Demographic characteristics | Weight | 53.6 [kg] | 109 [kg] |
|  | Age | 33 [years] | 47 [years] |
| Drug-specific parameter | CL_int,OAT1,3, in vitro_ | 1.95  [µl/min/mg protein](3) | 7.1  [µl/min/mg protein](4) |
|  | f_u, adult_ | 0.8(3) | 0.31(4) |
| CL_R_ values | Total CL_R_ (literature) | 13.6 [L/h](7) | 4.5 [L/h](6) |
|  | Activity adjustment factor (AAF) | 11.6 | 0.65 |

**Supplemental Material References**

1. Salem F, et al. A re-evaluation and validation of ontogeny functions for cytochrome P450 1A2 and 3A4 based on in vivo data. Clin Pharmacokinet. 2014;53(7):625–36.

2. Simcyp (a Certara Company). Simcyp v18. 2018.

3. Wen S, et al. OAT1 and OAT3 also mediate the drug-drug interaction between piperacillin and tazobactam. Int J Pharm. 2018;

4. Mathialagan S, et al. Quantitative prediction of human renal clearance and drug-drug interactions of organic anion transporter substrates using in vitro transport data: A relative activity factor approach. Drug Metab Dispos. 2017;

5. T’jollyn H, et al. Strategies for Determining Correct Cytochrome P450 Contributions in Hepatic Clearance Predictions: In Vitro–In Vivo Extrapolation as Modelling Approach and Tramadol as Proof-of Concept Compound. Eur J Drug Metab Pharmacokinet. 2017;

6. Brill MJE, et al. Reduced subcutaneous tissue distribution of cefazolin in morbidly obese versus non-obese patients determined using clinical microdialysis. J Antimicrob Chemother. 2014;69(3):715–23.

7. Butterfield JM, et al. Pharmacokinetics and pharmacodynamics of extended-infusion piperacillin/tazobactam in adult patients with cystic fibrosis-related acute pulmonary exacerbations. J Antimicrob Chemother. 2014;

**Figures**


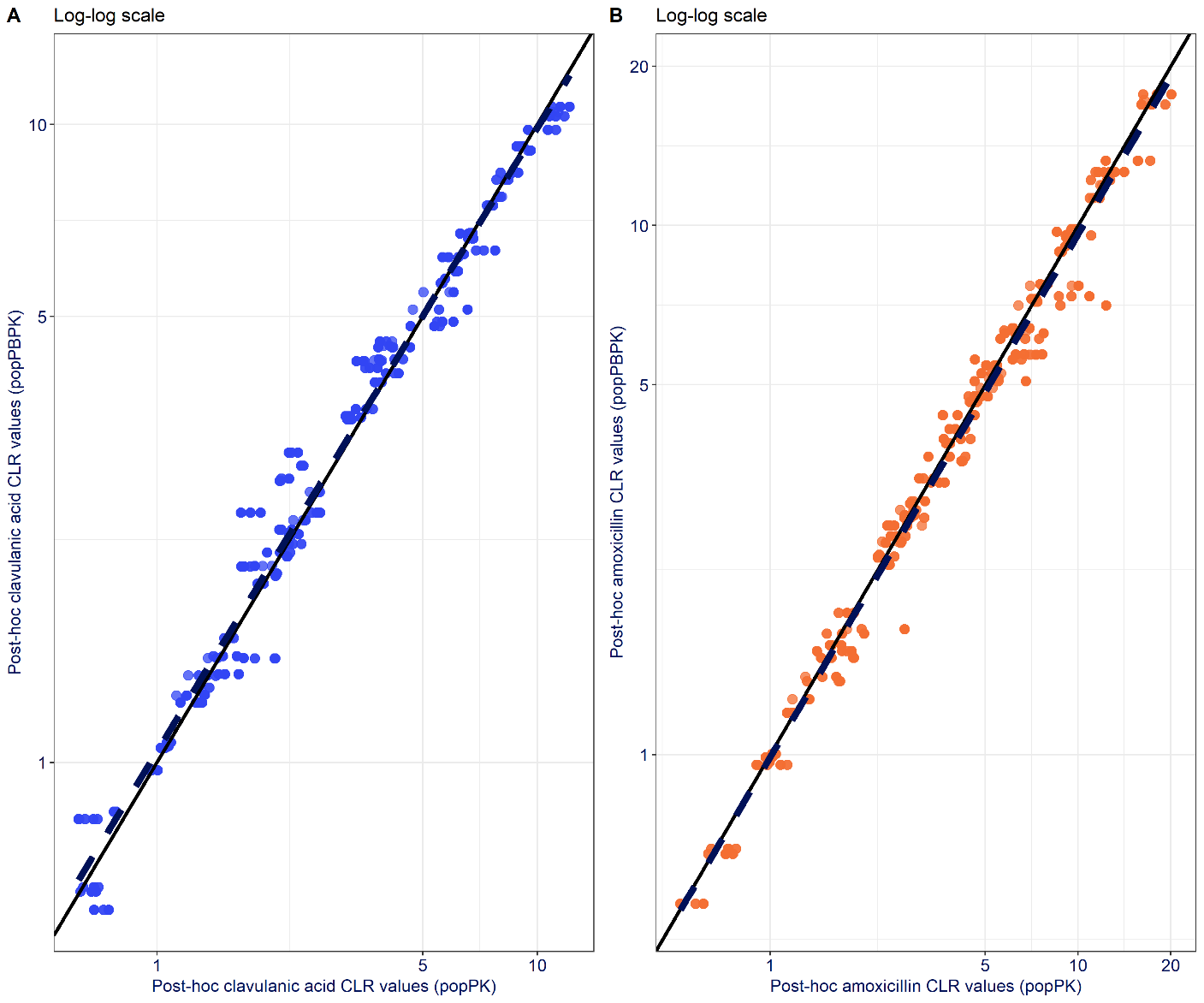


Figure S1 - Individual post-hoc CL_R_ predictions of clavulanic acid (left panel) and amoxicillin (right panel) obtained with the population PK approach vs. individual post-hoc CL_R_ predictions obtained with the combined population and PBPK approach (popPBPK). A line of identity (solid line) and a linear regression line (dashed line) are added to the graph. The data points are scattered around the line of identity without bias. Plots are on a double-log scale.


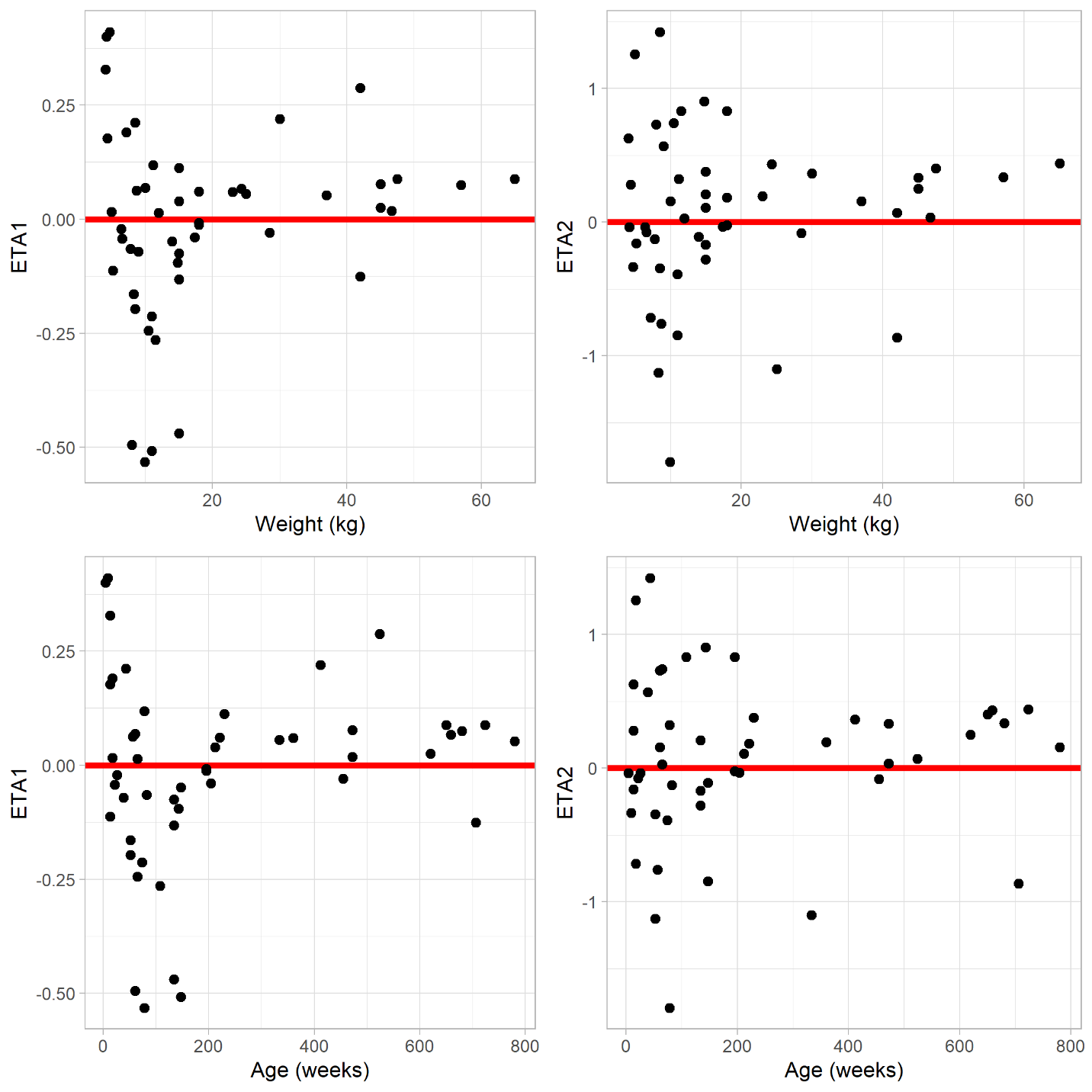


Figure S2- ETA vs. covariates correlation plots. This shows the correlation between ƞ_CLint,OAT1,3,in vivo_ (ETA1) and . ƞ_GFR_ (ETA2) as estimated with the popPBPK approach and weight and postnatal age in weeks, in the model including the ontogeny function for OAT1,3. Red line is the zero line, the theoretical mean of the ETAs.
